# Milk-Sickness

**Published:** 1867-06

**Authors:** L. P. Newman

**Affiliations:** Chicago, Ill.


### MILK-SICKNESS.

By L. P. Newman, M.D., Chicago, Ill.

An article on this subject, in a recent number of the Journal, recalls to my mind a series of observations made by myself, in the year 1865, upon an epidemic called Milk-Sickness, then prevailing in the "Western Reserve," in Ohio.

The surface of the infected district is undulating and woody, interspersed with low prairie or marsh lands, covered with water, grass, and low brush. The prevailing diseases are of the periodical type.

I first noticed the disease in milch cows; the characteristic symptoms being an appearance of great agony, with weakness of the knees and trembling of the whole frame; the bowels constipated, the fæces hardened; the urinary secretion entirely suspended. A speedy death the result. In examining the animal after death, the skin was found very much thickened, and covered with small pustules. The subcutaneous tissue was filled with serum, giving the bloated, anasarcous condition. The large viscera generally congested with very dark blood.

I have examined the bodies, not only of numerous kine, but of the horse, hog, sheep, and dog, also of fowls, both domestic and wild—especially the buzzard. In all my examinations, I found the same condition present. Whence animals receive this affection, I am unable to determine, but men procure it from eating the meat and drinking the milk of the infected. I have never seen a case from the first-mentioned cause, but hundreds from the latter. The symptoms were precisely the same as those noticed in animals, with the exception, that in man there was extreme nausea and vomiting. The skin was dry and hot; the tongue heavily coated upon a red body; the pulse very rapid; respiration difficult, causing much pain around the umbilicus; the abdomen inflated and tender.

It is generally fatal if neglected, but if, called early, before the impression of the poison upon the system became profound,

I could succeed in arresting the vomiting and loosen the bowels, my patient was safe. If relief was not near, the King of Terrors claimed his victim. One case, I will state:—

*May 18th.* Was called to see Mr. B., who had that morning, whilst in perfect health, drank some sweet milk from a *fresh* cow. One hour afterwards he was attacked with the symptoms just described. Ordered half an ounce syrup zingiberis, which checked the vomiting. Then directed pil. cathart. comp., three to be repeated every four hours. The following prescription was then written by my counsel, Dr. Johnston:—℞. Hydrarg. chlorid. mit. quiniæ sulph., āā gr. xxx. M. In chart. vj. divid. S. One every hour. The result was happy; our patient recovered. We pursued this course of treatment with great success.

In one *post mortem* of a human subject which I made, I found the same general morbid features noticed in similar examination of animals. The heart, lungs, and brain highly congested with dark, grumous blood.

So far as I am advised, "Milk-Sickness" is most prevalent in highly malarious districts—are not the cattle feeding in and inhabiting these localities affected by a strictly malarious fever, which, transmitted by this channel to the human being, assumes a malignant type of the same disease? Such is the impression I have myself received from observing it, and, if erroneous, I hope to be excused by a profession ever willing to recognize an honest purpose.

